# Sarcopenic Obesity and Cardiometabolic Health and Mortality in Older Adults: a Growing Health Concern in an Ageing Population

**DOI:** 10.1007/s11892-023-01522-2

**Published:** 2023-08-11

**Authors:** Sasiwarang Goya Wannamethee, Janice L. Atkins

**Affiliations:** 1 Department Primary Care and Population Health, University College London, Royal Free Campus, London, NW32PF UK; 2 Epidemiology and Public Health Group, Department of Clinical and Biomedical Sciences, Faculty of Health and Life Sciences, University of Exeter, St Luke’s Campus, Exeter, EX1 2LU Devon UK

**Keywords:** Sarcopenia, Obesity, Cardiovascular disease, Metabolic disease, Diabetes, Ageing

## Abstract

**Purpose of Review:**

Sarcopenic obesity (SO) is a growing public health problem in older adults. Whether SO confers higher risk of cardiometabolic disease and mortality than obesity or sarcopenia alone is still a matter of debate. We focus on recent findings on SO and cardiometabolic health and mortality in older adults.

**Recent Findings:**

SO is associated with increased mortality compared to non-sarcopenic obesity, but similar mortality risk compared to sarcopenia without obesity. SO is associated with a higher risk of cardiovascular disease (CVD), diabetes, and physical disability than obesity or sarcopenia alone. SO, in the presence of diabetes, is associated with the highest risk of CVD and chronic kidney disease. A definition and diagnostic criteria for SO has recently been proposed (ESPEN and EASO).

**Summary:**

SO is associated with more adverse outcomes overall than sarcopenia or obesity alone. Future research is required to assess the impact of the new SO definition on health outcomes.

## Introduction

Obesity is a major public health problem concern in all age groups with prevalence increasing worldwide [1, 2]. It is a well-established risk factor for metabolic diseases such as cardiovascular disease (CVD) and diabetes [3, 4] and is associated with morbidities that influences quality of life as well as life expectancy [5]. The number of adults aged 65 years or over, currently comprising 13% of the global population, is increasing worldwide and is projected to reach 21% of the global population (2.1 billion people) by 2050 [6]. As people age, there is a relative increase in visceral abdominal fat and a progressive loss of muscle strength and mass known as sarcopenia [7]. Visceral fat is an important contributor to the development of metabolic disorders including hypertension, dyslipidaemia, and insulin resistance and CVD and diabetes [3, 4]. Sarcopenia is associated with metabolic impairments including insulin resistance as well as CVD risk factors [7–9]. Sarcopenia is also associated with increased risk of falls, fractures, physical disability hospitalization, and mortality [7–11] and predicts loss of independent daily life activities in the elderly [12]. A number of pathological mechanisms underlying age-related muscle loss have been identified including neuronal and hormonal changes, being underweight, poor nutrition, physical inactivity, insulin resistance and inflammation [13–20]; thus, sharing many pathological mechanisms with atherosclerosis [14, 20]. Sarcopenia is highly prevalent in aging populations affecting 30% of people over the age of 60 and more than 50% of those over the age of 80 and is now a recognised major health issue in the elderly [12]. Sarcopenia is often associated with visceral fat; the confluence of ageing with rising obesity rates has led to a phenomenon termed sarcopenic obesity (SO) defined as the combination of sarcopenia and obesity [7, 21]. Many recent reviews have highlighted SO as a growing public health burden [22–24, 25•, 26•]. SO is correlated with multiple adverse cardiometabolic effects and is associated with many poor health outcomes such as frailty, falls, disability, and increased morbidity and mortality [22–24, 25•, 26•] as well as complications in those with disease conditions such as diabetes [27]. Moreover, sarcopenia is recognized as a new complication in elderly patients with type 2 diabetes mellitus (T2DM) [28].

Many studies to date suggest that older adults with SO have higher rates of CVD and an increased mortality risk compared with those without sarcopenia or obesity and it is suggested that having both obesity and sarcopenia together may present an even greater risk for adverse health outcomes and mortality in the elderly than having either obesity or sarcopenia alone [14, 25•, 29] although this is still debatable [22]. Despite the health significance of SO, it remains poorly managed in clinical practice because of the different definitions and lack of universal consensus in defining SO diagnostic criteria [30]. The European Society for Clinical Nutrition and Metabolism (ESPEN) and the European Association for the Study of Obesity (EASO) recognize SO as a scientific and clinical priority. Consequently, the ESPEN AND EASO have recently published the first consensus definition and diagnostic criteria for SO [31••]. In this article, we review the evidence that SO is associated with higher risk of mortality and metabolic diseases than obesity or sarcopenia alone as well as the impact of SO on CVD and related morbidity in the presence of T2DM. We also discuss the definition, diagnostic criteria, and preventive approach for SO in the elderly.

## Definition and Diagnosis of Sarcopenic Obesity

### Sarcopenic Obesity

**Fig. 1:**
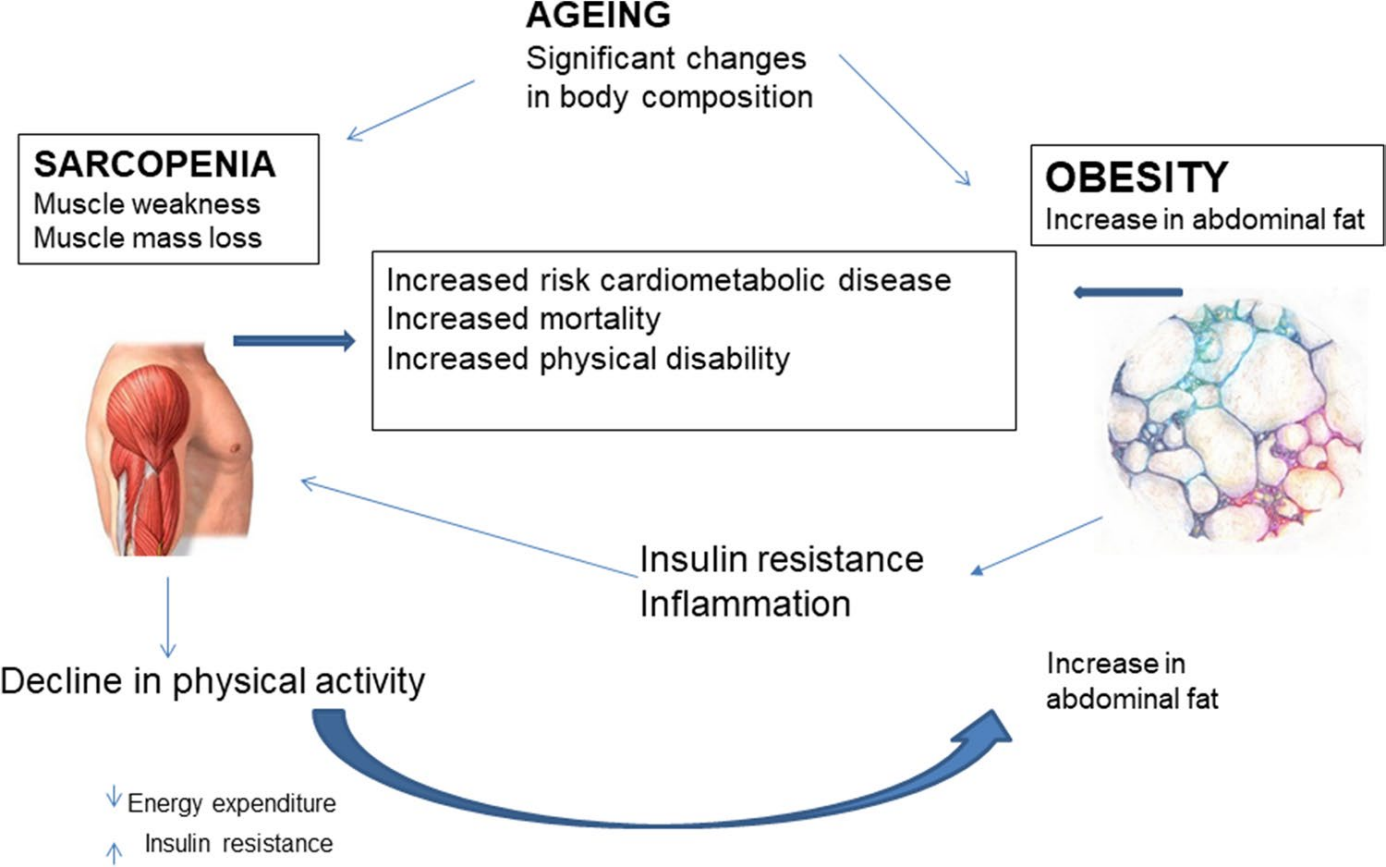
Interplay between sarcopenia and obesity in ageing. Adapted from Wannamethee et al. 2015 [29]

Ageing is associated with significant changes in body composition with a decrease in muscle mass and an increase in visceral fat. Visceral fat and muscle mass are known to be interrelated pathogenetically and are reported to share common inflammatory pathways [14]. Sarcopenia can lead to reduced physical activity which in turn can lead to decreased energy expenditure resulting in increased risk of obesity [14]. Alternatively, visceral fat could increase inflammation leading to the development of sarcopenia [19]^.^ The term ‘sarcopenic obesity’ was first coined by Baumgartner et al. [7] characterized as a combination of sarcopenia (loss of muscle mass and function), and obesity (excess fat mass) (Fig. 1). SO has emerged as a new category of obesity and an important public health concern in the elderly [14]. Obesity and sarcopenia are both associated with metabolic disorders, morbidity, disability, and mortality [14]. Individuals with SO may have greater risk of metabolic abnormalities, CVD, and mortality than those with obesity or sarcopenia alone.

The criteria cut-offs used in previous studies to define both sarcopenia and obesity have varied widely depending on the population, age group, gender, and ethnicity. In a review of 50 studies including patients ≥ 60 years, it is estimated that globally, SO affects more than 1 in 10 adults aged > 60 [32]. The prevalence increases steeply with age to almost 1 in 4 older adults aged > 75 years, but there is considerable variation in the prevalence due to different definitions and age groups and ethnic groups used across studies.

### Diagnostic Criteria for Sarcopenic Obesity

In 2010, the European Working Group on Sarcopenia in Older People (EWGSOP) published consensus guidelines on the definition and diagnosis of sarcopenia [33]. This was based on the presence of impaired physical performance using indicators of slow gait speed, followed by either the presence of low muscle mass measured using methods such as dual energy X-ray absorptiometry (DXA), bioimpedance analysis (BIA) computed tomography (CT) and magnetic resonance imaging (MRI) or presence of low muscle strength (e.g., assessed with hand grip strength). In 2014, the Foundation for the National Institutes of Health (FNIH) Sarcopenia Project recommended an alternative sarcopenia definition using gender specific cut-off points for appendicular lean mass and grip strength [34]. In 2019, an updated sarcopenia definition was published by the EWGSOP (the EWGSOP-2) which focused on low muscle strength as a key measure of sarcopenia. Detection of low muscle quantity and quality was used to confirm the diagnosis, and poor physical performance was used to identify severe sarcopenia [35]. A joint ESPEN and EASO expert panel recently published the first consensus definition and diagnostic criteria for SO which could be implemented in general practice. The panel proposed that SO be defined as the presence of both excess adiposity and low muscle mass or strength. The ESPEN and EASO SO criteria consist of three steps: screening, diagnosis, and severity grading [31••]. The diagnosis of SO is considered when the individual screens positive for elevated body mass index or waist circumference, and markers of low skeletal muscle mass and function based on risk factors, clinical symptoms, or validated questionnaires. The recommended diagnostic procedures include assessment of skeletal muscle function using handgrip strength or the chair stand test, followed by assessment of body composition using DXA or bioelectric impedance analysis. Individuals with SO are further stratified into Stage I where there are no clinical complications of sarcopenia, or Stage II if cases are associated with complications associated with sarcopenia.

### Consequences of Sarcopenic Obesity

#### Mortality

Whether SO has additive mortality risk over having sarcopenia or obesity alone is still a matter of debate [22]. Findings from a meta-analysis of 23 prospective cohorts including 50,866 participant aged 50–82.5 years showed that SO was associated with a significant 21% increase in mortality compared with those without SO, hazard ratio (HR) = 1.21 (95% CI = 1.10–1.32) [36]. However, the meta-analysis did not assess the importance of the two components of SO individually (sarcopenia vs obesity).

Most recent prospective studies have not shown adults with SO to have higher mortality risk than those with sarcopenia only. In a pooled analysis of 4,612 participants aged 70 years or more from three prospective cohorts (Health 2000, Health ABC, and the Longitudinal Aging Study Amsterdam), [37] probable sarcopenia, whether combined with obesity or not, is associated with increased mortality compared to those with no probable sarcopenia and no obesity. However, those with probable sarcopenia and obesity did not show higher risk than those with probable sarcopenia alone. In the English Longitudinal Study of Ageing (ELSA) (60 + years), SO based on grip strength and BMI did not confer greater mortality than sarcopenia alone [38]. In the Honolulu Heart Study of 2,309 elderly men aged 71 to 93, sarcopenia and SO as defined by waist circumference (but not BMI) and skeletal muscle mass was associated with increased mortality compared to the normal group. Obesity alone was not associated with increased mortality [39]. In the UK Biobank study of over 400,000 subjects, age 40–70 years, those with SO showed higher mortality than those with obesity alone but individuals with SO had estimated mortality risks comparable to individuals with sarcopenia without obesity. Compared to the non-obese and non-sarcopenic group, the hazard ratios (95% CI) for total mortality were 1.14 (1.08–1.21) for obesity alone, 1.39 (1.30–1.48) for sarcopenia alone and 1.31 (1.18–1.45) for SO [40]. By contrast, in a study of over 4,000 men aged 60–79 years (the British Regional Heart Study), mortality risk was the highest in men with sarcopenia and obesity defined on the basis of high waist circumference and low midarm muscle circumference [41]. Compared with the normal group (without sarcopenia or obesity), mortality risk was increased by 41% in men with sarcopenia, 21% in men with obesity and 72% in men with sarcopenia and obesity after adjustment for potential confounders [41]. However, not all studies have shown SO to be associated with mortality. In the National Health and Nutrition Examination Survey of adults aged over 70, SO defined as combined low muscle mass and body fat percentage showed no association with mortality [42]. These inconsistencies may be due to more advanced age and the definition of sarcopenia (muscle quantity or quality assessments). The EWGSOP2 recognises low muscle strength more than low muscle mass to be the principal determinant of sarcopenia [35]. A recent systematic review and pooled meta-analysis of SO on mortality by Liu et al. which included over 167,000 elderly subjects (mean age 70.6 years) indicated that subjects with SO had higher mortality than subjects with obesity alone, but risk was comparable to those with sarcopenia only. The hazard ratios and 95% CI were 1.51 (1.14–2.02) for SO, 1.49 (1.27–1.75) for sarcopenia and 1.02 (0.86–1.23) for obesity alone [43••]. To date there is yet no study on the impact of SO diagnosed using the ESPEN and EASO consensus definition.

### Cardiometabolic Risks of Sarcopenic Obesity

#### Metabolic Risk Factors

Both sarcopenia and obesity are each associated with metabolic disorders and in combination may exert harmful effects on metabolic and cardiovascular health compared with obesity or sarcopenia alone. A review of observational studies on the association between SO and CV risk factors indicates that SO is associated with metabolic abnormalities, inflammation, and CVD risk factors and many but not all studies show older adults with SO to have the highest level of cardiovascular risk factors including metabolic syndrome, hypertension, dyslipidaemia, insulin resistance and inflammation [25•]. A recent meta-analysis that included 106 observational studies comprising a total of 167,151 elderly patients showed sarcopenic obesity and non-sarcopenic obesity to have similar risk of hypertension, dyslipidaemia, and the metabolic syndrome but both groups with obesity showed higher risk than those with sarcopenia alone [43••].

#### Cardiovascular Disease (CVD)

Several prospective studies have investigated the association between sarcopenic obesity and incident CVD with inconsistent results [25•]. In the Cardiovascular Health Study of over 3,000 older adults aged 56 and above, SO, defined using waist circumference and BIA-measured muscle mass, was not associated with significantly increased risk of incident CVD when compared to the normal group. However, the group with SO showed the highest risk of CVD when SO was defined using grip strength and waist circumference [44]; risk of CVD was 23% higher compared to the normal group. In the British Regional Heart Study of over 4000 men aged 60–79 years, no association was seen between SO (defined by midarm muscle circumference and waist circumference) and CVD events or CVD mortality; this study did not examine muscle strength measures [41]. The evidence from these studies suggests that muscle strength could be a better measure of sarcopenia than muscle mass in predicting CVD risk which is consistent with EWGSOP2 recognising low muscle strength more than low muscle mass to be associated with sarcopenia. More recently, in the large UK Biobank prospective study of over 400,000 subjects, age 40–70, SO (defined by hand grip strength and BMI) was associated with the highest risk of CVD events and CVD mortality, compared to the other three body composition categories, among 40- to 70-year-olds [40]. When compared to subjects without obesity or sarcopenia, the hazard ratios (95% CI) for CVD events were 1.29 (1.24–1.35) for obesity alone, 1.04 (0.98–1.11) for sarcopenia alone and 1.42 (1.31–1.55) for the group with SO; for CVD mortality, the corresponding hazard ratios were 1.46 (1.30–1.63), 1.67 (1.45–1.92) and 1.78 (1.45–2.18), respectively. The inconsistencies seen between prospective studies are perhaps due to the varying definitions used to define sarcopenia and SO. A recent meta-analysis that included over 167,000 elderly subjects also showed SO to be associated with the highest risk of CVD and coronary artery disease (CAD) events; risk was higher compared to obesity or sarcopenia alone. For CVD events, the odds ratio (OR) was 1.19 (0.40–1.98) in subjects with obesity without sarcopenia, 1.51 (1.00–2.29) in those with sarcopenia only, and 1.97 (1.25–3.11) in the group with SO; for CAD events, the corresponding ORs were 1.17 (0.86–1.58), 1.85 (1.41–2.44) and 2.48 (1.85–3.31), respectively [43••].

#### Type 2 Diabetes (T2D)

While obesity is a major causal risk factor for T2D, sarcopenia may also contribute to the development of diabetes through altered glucose disposal due to low muscle mass and increased local inflammation [45, 46]. A few prospective studies have shown that low muscle mass and low muscle strength are associated with increased risk of incident T2D [47, 48]. Less has been studied on the association between SO and risk of T2D. A recent systematic review of 11 cross-sectional studies including a total of 60,118 adults with overweight or obesity indicated that SO increases the risk of T2D by nearly 38% (OR [95%CI]; 1.38 [1.27–1.50]) compared to those with overweight or obesity alone [49], although the results should be interpreted with caution because of the cross-sectional nature of the studies. In a recently published retrospective longitudinal study from Korea, 36,304 diabetes-free adults (age 48.9 ± 8.8 years) were classified into 4 groups of normal, presarcopenia alone, abdominal obesity alone, and presarcopenic obesity according to initial body composition based on waist circumference and appendicular skeletal muscle mass measured by BIA [50]. Compared to normal body composition, presarcopenia and obesity alone were each associated with a greater risk of incident T2D (adjusted HR [95% CI]; 1.31 [1.18–1.45] and 1.37 [1.24–1.51], respectively); presarcopenic obesity was associated with the highest risk (1.57 [1.42–1.73]).

## Sarcopenic Obesity and Health Outcomes in Patients with type 2 Diabetes

In patients with diabetes, the incidence of sarcopenia is 2–3 times higher compared to patients without diabetes [28]. A number of studies have examined the association between SO and CVD and mortality outcomes in patients with diabetes. In a retrospective Chinese study of 386 elderly patients with T2D [51], the hazard ratios (95% CI) for all-cause mortality or fragility fracture were 1.28 (0.58–2.86) in patients with nonsarcopenic obesity, 1.81 (0.63–5.20) in those with sarcopenic nonobesity, and 2.94 (1.25–6.92) in those with SO, compared to patients with nonsarcopenic nonobesity. For incident CVD, the corresponding hazard ratios were 4.28 (1.22–14.99), 2.63 (0.55–12.50) and 6.02 (1.56–23.15), respectively. In a study of 716 Japanese patients with diabetes (mean age 65 ± 13 years), SO assessed using DXA predicted incident CVD [52]. The hazard ratios (95%CI) were 1.88 (0.75–4.75) for sarcopenia only, 0.98 (0.44–2.21) for obesity only, and 2.63 (1.10–6.28) for SO.

Diabetes is a major cause of chronic kidney disease (CKD) [53]. There is mounting evidence that sarcopenia is associated with CKD [54, 55] and some studies have indicated a strong link between SO and CKD in patients with T2D. In a study of 745 people with T2D (mean age 64.6 years, 53.6% men), SO was associated with > 30% decline in estimated glomerular filtration rate (eGFR) in both men and women and showed higher rates of decline than those with sarcopenia or obesity alone [56]. In a retrospective longitudinal cohort study of 3,123 patients with T2DM, SO defined by skeletal muscle mass index and waist circumference was independently associated with an increased risk of incident CKD [57]. SO was associated with a 70% increase in risk of developing CKD (HR [95% CI]; 1.77 [1.24–2.51]) compared with a HR (95%CI) of 1.04 (0.78–1.38) for sarcopenia alone and 1.27 (0.75–2.14) for obesity alone. It was suggested that assessment of WC and sarcopenia may serve as a tool to identify those at high risk of CKD in patients with T2DM.

### Functional Limitation

There is strong evidence that SO confers higher risk of physical disability than obesity or sarcopenia alone. The recent meta-analysis including over 167,000 elderly subjects showed those with SO to have higher risk of functional limitation than those with sarcopenia or obesity alone. The OR (95%CI) was 2.92 (2.12–4.02) for SO, 1.89 (1.40–2.56) for sarcopenia only and 1.20 (0.88–1.65) for obesity only [43••].

## Screening for Sarcopenia in Primary Care

Targeting obesity and sarcopenia may provide an effective approach for preventing metabolic diseases and increasing functionality. Although there has been much research on sarcopenia over the last few decades, systematic screening for sarcopenia is not commonly carried out in geriatric care. Due to the adverse effects of sarcopenia on health the ESPEN and the EASO have recently published a consensus definition and diagnostic criteria for SO which could be implemented in screening for SO in general practice [31••]. Screening is based on the presence of elevated BMI or waist circumference together with markers or indicators of sarcopenia which includes a list of chronic diseases, weight loss, weakness, exhaustion, and movement limitations. If there is suggestion of the presence of sarcopenia based on these measures, the diagnostic procedure will proceed to assessment of body composition by DXA or BIA, and skeletal muscle strength or chair stand test. Screening older people with evidence of low physical performance using simple questions based on presence of chronic diseases weight loss and self-reported mobility limitation that do not require performance testing or complex methods for body composition measurements may help identify older adults with early evidence of sarcopenia who would benefit from intervention. This may be a practical first approach in primary care to target and prevent sarcopenia where measuring devices for muscle mass or strength performance are not commonly available in routine clinical practice.

## Lifestyle and Therapeutic Intervention

Physical exercise and nutrition are important lifestyle factors that contribute to sarcopenia, obesity and SO [58–61]; optimal diet and exercise strategies are likely to play a key role in preventing and managing SO. A systematic review of studies on exercise intervention for improving performance in adults with SO concluded that physical exercise and in particular resistance training can improve or maintain physical performance in adults with SO [60]. In the Louisiana Osteoporosis Study, it was suggested that regular exercise >  = 3/week was associated with lower risk of having obesity and SO in older males and females [62]. Diets lacking in protein have been shown to influence the development of sarcopenia [59] and the European Society for Clinical Nutrition and Metabolism Expert Group have recommended protein intake of 1.0–1.2 g/kg bodyweight/day to help maintain muscle mass and strength in older adults over 65 years [63]. Other therapeutic interventions and therapies available for SO have been reviewed and include gene therapy, anabolic hormones, and antioxidants [23].

## Conclusion

SO is prevalent in older people and is associated with numerous cardiometabolic disorders and adverse health outcomes including increased mortality, metabolic diseases and functional limitation compared to adults who are not obese and who do not have sarcopenia. The pathogenesis is multifactorial, but it seems that inflammatory mediators and insulin resistance play an important role. People with SO have a worst metabolic risk profile than those with obesity alone. Whether SO confers a higher risk of mortality compared to obesity or sarcopenia alone is still debatable but there is accumulating evidence that older individuals with SO have higher risk of mortality than those with obesity alone, but risk is not higher than in those with sarcopenia only. By contrast, there is mounting evidence that SO is associated with higher risk of cardiometabolic disease and physical disability than obesity or sarcopenia alone. SO, in the presence of diabetes, is associated with the highest risk of adverse CV outcomes and CKD. SO is a potentially modifiable condition; hence there is a need to prevent and manage SO. Efforts to improve healthy ageing should focus on preventing obesity and maintaining muscle mass and strength in older adults. It is likely that the combination of optimal diet and exercise intervention are effective strategies for preventing and managing sarcopenia and SO. The joint consensus statement of the ESPEN and EASO has provided a definition of sarcopenic obesity and a three-stage procedure to screen and diagnose sarcopenia in the elderly. Further research on the impact of SO diagnosed using ESPEN and EASO definitions on health outcomes is needed and to identify the best therapeutic approaches for sarcopenic obesity.
